# Crystal structure, Hirshfeld surface and photo­physical analysis of 2-nitro-3-phenyl-9*H*-carbazole

**DOI:** 10.1107/S2056989021007726

**Published:** 2021-08-03

**Authors:** Neetu Singh, Ga Hee Noh, Hyoung-Ryun Park, Junseong Lee

**Affiliations:** a Department of Chemistry, Chonnam National University, Gwangju, 61186, Republic of Korea

**Keywords:** crystal structure, carbazole, Cadogan reaction

## Abstract

The title compound, C_18_H_12_N_2_O_2_, was synthesized from a di­nitro­biphenyl­benzene derivative using a novel modification of the Cadogan reaction. The reaction has several possible ring-closed products and the title compound was separated as the major product. The X-ray crystallographic study revealed that the carbazole compound crystallizes in the monoclinic *P*  space group and possesses a single closed Cadogan ring. There are two independent mol­ecules in the asymmetric unit. In the crystal, the mol­ecules are linked by N—H⋯O hydrogen bonding.

## Abstract 2

The title compound was synthesized from a di­nitro­biphenyl­benzene derivative using a novel modification of the Cadogan reaction. The reaction has several possible ring-closed products and the title compound was separated as the major product. It crystallizes in the monoclinic *P* 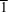 space group and possesses a single closed Cadogan ring.

## Chemical context   

Carbazole consists of two benzene ring fused on either side of a central pyrrole ring and is also known as dibenzo­pyrrole or di­phenyl­enimine. This N-containing heterocyclic compound was discovered by Graebe and Glaser in 1872 (Collin *et al.*, 2006 ▸). Carbazoles represent an important class of heterocycles with several advantages. By the introduction of substituents in the carbazole fragment at the nitro­gen atom and the aromatic framework at positions 3 and 6, the photophysical properties can be modified (Srivastava & Chakrabarti, 2017 ▸; Sun *et al.*, 2015 ▸). The high stability and redox potential property of carbazole-based polymers compared with other conducting polymers has attracted a great attention (Nandy *et al.*, 2014 ▸; Bashir *et al.*, 2015 ▸; Sutanto *et al.*, 2021 ▸; Niu *et al.*, 2021 ▸). Carbazole-based ligands exhibit high hole-transporting mobility and strong absorption in the UV–visible spectroscopic region, and therefore show good electro- and photoactive properties (Yavuz *et al.*, 2001 ▸). Polycyclic compounds containing two pyrrole rings have become widely used because of their good charge-transfer properties and the feasibility of tuning the electronic levels in the compound for different types of applications (Wakim *et al.*, 2008 ▸; Reig *et al.*, 2015 ▸; Xiang *et al.*, 2018 ▸; Zhang *et al.*, 2018 ▸; Szafraniec-Gorol *et al.*, 2021 ▸), These types of compounds are therefore excellent candidates for applications such as OLEDs (organic light-emitting diodes; Svetlichnyi *et al.*, 2010 ▸; Oda *et al.*, 2021 ▸; Zhou *et al.*, 2021 ▸; Bao *et al.*, 2020 ▸), DSSCs (dye-sensitized solar cells; Zhang *et al.*, 2009 ▸; Li *et al.*, 2018 ▸; Lokhande *et al.*, 2019 ▸), OPV (organic photovoltaics; Chan *et al.*, 2013 ▸; Yang *et al.*, 2020 ▸) and OFETs (organic field-effect transistors; Reig *et al.*, 2015 ▸; Chen *et al.*, 2020 ▸; Koli *et al.*, 2020 ▸).

The title compound was isolated as an inter­mediate in the middle of the synthetic route for the synthesis of double Cadogan-fused carbazoles. The reaction between 1,3-di­nitro­diphenyl­benzene and tri­phenyl­phosphine using the solvent *o*-di­chloro­benzene resulted in a mixture of single- and double-Cadogen ring-closure products. First, a di­nitro compound was obtained by a nitration reaction and in the second step, performing double Suzuki coupling reaction on 1,5-di­bromo-2,4-di­nitro­benzene and benzene­boronic acid gave a terphenyl compound. Then, in the final step, a single Cadogan ring closure was performed to obtain the title compound, **1**.
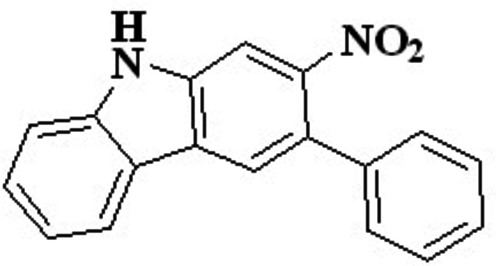


## Structural commentary   

**Figure 1:**
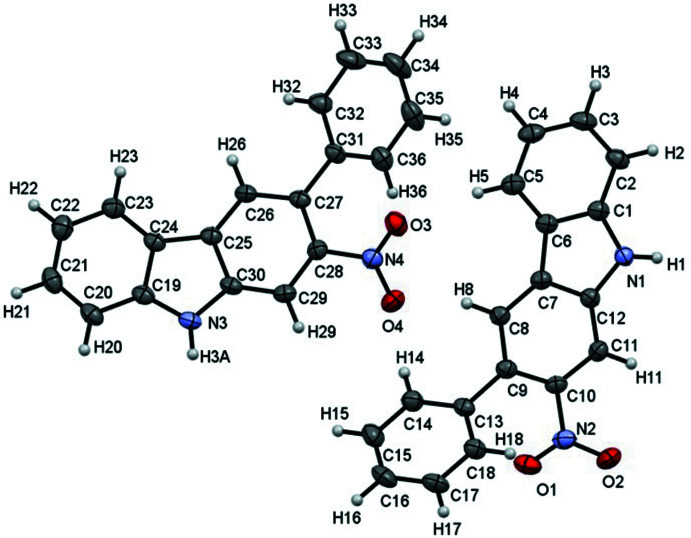
The asymmetric unit of the title compound, with atom labelling and displacement ellipsoids drawn at the 50% probability level.

Structural analysis confirmed the formation of a single Cadogan ring major product, *i.e*. carbazole with a nitro group at the 2-position, and a phenyl group at the 3-position. The mol­ecular structure of compound **1** is shown in Fig. 1 ▸. There are two independent mol­ecules in the asymmetric unit in which the dihedral angles between the carbazole ring system (r.m.s. deviations of 0.001 and 0.002 Å for the N1-carbazole and N3-carbazole units, respectively) and the attached phenyl rings are 55.54 (6) and 43.46 (7)°.

## Supra­molecular features   

**Figure 2:**
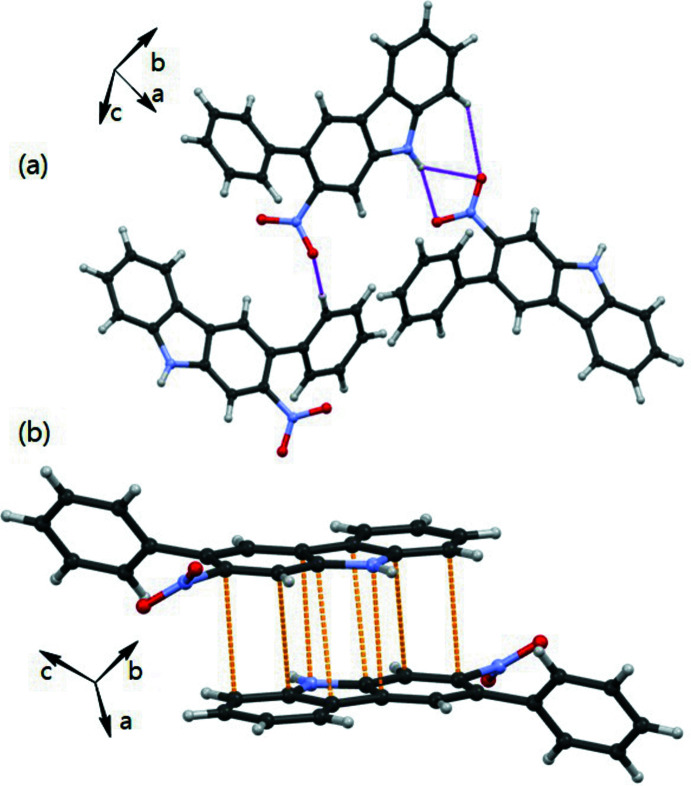
A plot showing (*a*) the inter­molecular N—H⋯O, C—H⋯O hydrogen bonds and (*b*) π–π inter­actions.

In the crystal, the two mol­ecules are linked into [110] chains by N—H⋯O and N—H⋯N hydrogen bonds involving the carbazole N atom of one independent mol­ecule and the nitro group of the other (Table 1 ▸), as shown in Fig. 2 ▸. In addition, π–π stacking inter­actions occur along the *c*-axis direction [*Cg*1⋯*Cg*1(1 − *x*, −*y*, 1 − *z*) = 3.3963 (9) Å and *Cg*8⋯*Cg*8(1 − *x*, −*y*, −*z*) = 3.3982 (10) Å where *Cg*1 and *Cg8* are the centroids of the N1/C1/C6/C7/C12 and N3/C19/C24/C25/C30 rings, respectively] with adjacent carbazole rings within the stacks being almost parallel. The combination of hydrogen bonding and π-stacked carbazole ring systems results in the formation of a three-dimensional inter­action.

## Database survey   

A search of the Cambridge Structural Database (CSD Version 5.42, November 2020; Groom *et al.* 2016 ▸) using a fragment composed of carbazole with a nitro group gave only one hit, which did not have much in common with the title compound. The most similar reported compound is ABEPON (9-ethyl-3-methyl-1,6-di­nitro­carbazole; Asker *et al.*, 2004 ▸), whose main component consists of a nitro group on the carbazole ring. Examples of carbazole compounds substituted in the 3-position include ABAFOA (9-*p*-tolyl-9*H*-carbazole-3-carbo­nitrile; Ramathilagam *et al.*, 2011 ▸), ADALOH [3,6-di­bromo-9-(4-tolyl­sulfon­yl)-9*H*-carbazole; Li *et al.*, 2006 ▸], ANUWUD (dimethyl 9*H*-carbazole-1,3-dicarboxalate; Verma *et al.*, 2015 ▸) and ATAWEZ [3,6-dimeth­oxy-9-(2-tri­fluoro­meth­yl)phenyl-9*H*-carbazole; Matsubara *et al.*, 2016 ▸].

## Hirshfeld surface analysis   

**Figure 3:**
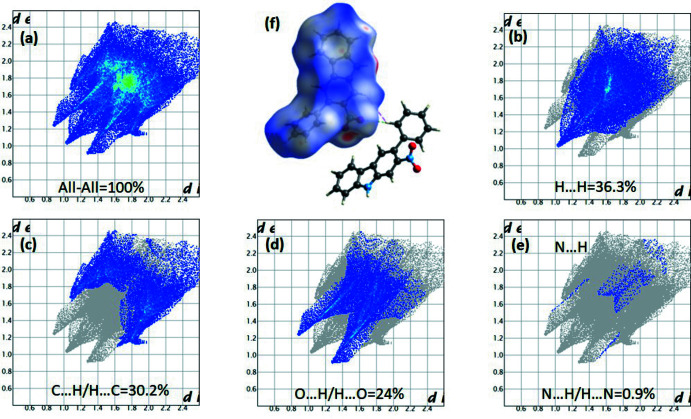
The Hirshfeld surface of the title compound mapped over *d*_norm_to visualize the inter­molecular inter­actions.

A Hirshfeld surface analysis (McKinnon *et al.*,2007 ▸; Spackman & Jayatilaka *et al.*, 2009 ▸) of compound **1** was performed with *CrystalExplorer17* (Turner *et al.*, 2017 ▸) to give an insight into the inter­molecular inter­actions. The Hirsfeld surface was calculated using a standard (high) surface resolution with the three-dimensional *d*_norm_ surface plotted over a fixed colour scale of −0.1339 (red) to 1.4773 a.u. (blue) as shown in Fig. 3 ▸. The red spots indicate short contacts, *i.e.* negative *d*_norm_ values on the surface, which highlight the hydrogen-bonding inter­actions.

The 2D finger plots shown in Fig. 3 ▸ indicate that the most important contributions to the overall surface are from H⋯H (36.3%), C⋯H/H⋯C (30.2%) and O⋯H/H⋯O (24%) inter­actions whereas the contribution of N⋯H/H⋯N inter­actions is almost negligible at 0.9%.

## Photophysical study   

**Figure 4:**
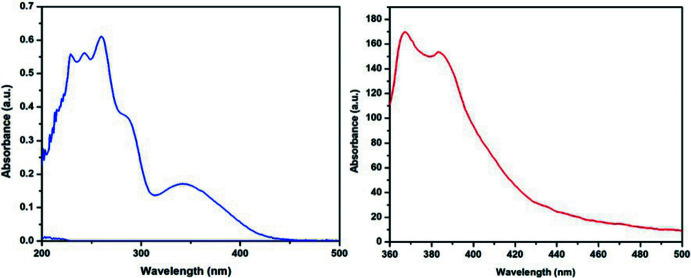
Absorption and emission spectra of the title compound **1** in DCM. The emission spectrum was excited at 350 nm.

The absorption and emission spectra of compound **1** were measured in dilute CH_2_Cl_2_ solution at room temperature, as shown in Fig. 4 ▸. Compound **1** exhibits an absorption band at 260 nm to 410 nm, which can be assigned to the carbazole moieties. The broad absorption bands at the lower energy peak around 350 nm suggest the formation of the carbazole dimer excimer from the carbazole groups. The PL spectrum of compound **1** excited at 350 nm shows a dominant blue–violet broad peak at 400 nm associated with the emission from the carbazole excimer.

## Synthesis and crystallization   

**Figure 5:**
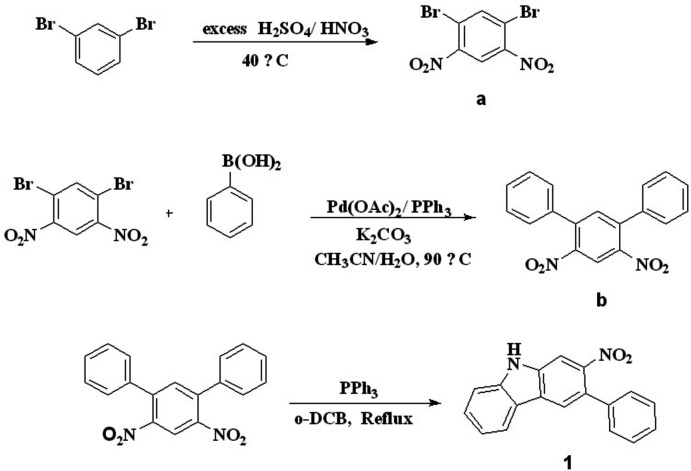
Reaction scheme.

The synthesis of the title compound is shown in Fig. 5 ▸. The reaction yielded single and double Cadogan ring-closure products. First we prepared di­nitro compound **a** by a nitration reaction and then we synthesized terphenyl compound **b** by performing double Suzuki-coupling reaction on 1,5-di­bromo-2,4-di­nitro­benzene and benzene­boronic acid. A two-necked flask fitted with a condenser was charged with 1,3-di­nitro-4,6-diphenyl benzene (**b**) (0.320 g, 1 mmol) and 0.655 g (2.5 mmol) of tri­phenyl­phosphine. 8 mL of the solvent *o*-di­chloro­benzene were added o the reaction mixture. The resulting reaction mixture was stirred at 473 K under nitro­gen for 24 h. The solvent was removed under reduced pressure at 333 K and the crude product was purified by column chromatography (silica gel, 10% EA in hexa­nes as eluent) to provide 0.230 g of the title product as a beige solid (yield: 86%). ^1^H NMR (500 MHz, CDCl3): δ 8.39 (*s*, 1H), 8.09 (*d*, *J* = 8.2 Hz, 1H), 8.05 (*d*, *J* = 9.9 Hz, 2H), 7.56–7.51 (*m*, 2H), 7.48–7.38 (*m*, 5H), 7.32 (*ddd*, *J* = 8.0, 6.4, 1.7 Hz, 1H).

## Refinement   

Crystal data, data collection and structure refinement details are summarized in Table 2 ▸. N-bound H atoms were refined with *U*_iso_(H) = 1.2*U*_eq_(N). C-bound H atoms were positioned geometrically (C—H = 0.95 Å) and refined as riding with *U*_iso_(H) = 1.2*U*_eq_(C).

## Supplementary Material

Crystal structure: contains datablock(s) I. DOI: 10.1107/S2056989021007726/ex2047sup1.cif

Structure factors: contains datablock(s) I. DOI: 10.1107/S2056989021007726/ex2047Isup2.hkl

Supporting information file. DOI: 10.1107/S2056989021007726/ex2047Isup3.cdx

Supporting information file. DOI: 10.1107/S2056989021007726/ex2047Isup4.cdx

Supporting information file. DOI: 10.1107/S2056989021007726/ex2047Isup5.cml

CCDC reference: 2099768

Additional supporting information:  crystallographic information; 3D view; checkCIF report

## Figures and Tables

**Table 1:** Hydrogen-bond geometry (Å, °)

*D*—H⋯*A*	*D*—H	H⋯*A*	*D*⋯*A*	*D*—H⋯*A*
N1—H1⋯O3^i^	0.88 (1)	2.33 (1)	3.1825 (16)	162 (1)
N1—H1⋯O4^i^	0.88 (1)	2.38 (1)	3.1331 (17)	143 (1)
N1—H1⋯N4^i^	0.88 (1)	2.59 (1)	3.4610 (17)	168 (1)
N3—H3*A*⋯O1^ii^	0.88 (1)	2.26 (1)	3.1079 (18)	162 (1)
N3—H3*A*⋯O2^ii^	0.88 (1)	2.45 (1)	3.2039 (19)	143 (1)
N3—H3*A*⋯N2^ii^	0.88 (1)	2.60 (1)	3.4700 (19)	170 (1)

Symmetry codes: (i) -x+1, -y, -z+1; (ii) -x+2, -y+1, -z+1.

**Table 2:** Experimental details

Crystal data	
Chemical formula	C_18_H_12_N_2_O_2_
*M*_r_	288.30
Crystal system, space group	Triclinic, *P*\overline{1}
Temperature (K)	100
*a*, *b*, *c* (Å)	9.2660 (3), 12.9590 (4), 13.1010 (4)
α, β, γ (°)	96.2487 (15), 109.1813 (15), 106.1061 (14)
*V* (Å^3^)	1392.39 (8)
*Z*	4
Radiation type	Mo *K*α
μ (mm^−1^)	0.09
Crystal size (mm)	0.1 × 0.1 × 0.1

Data collection	
Diffractometer	Bruker APEXII CCD
Absorption correction	Multi-scan (*SADABS*; Bruker, 2014 ▸)
*T*_min_, *T*_max_	0.628, 0.745
No. of measured, independent and observed [*I* > 2σ(*I*)] reflections	17357, 5277, 4470
*R*_int_	0.026
(sin θ/λ)_max_ (Å^−1^)	0.611

Refinement	
*R*[*F*^2^ > 2σ(*F* ^2^)], *wR*(*F* ^2^), *S*	0.041, 0.115, 1.08
No. of reflections	5277
No. of parameters	398
H-atom treatment	H-atom parameters constrained
Δρ_max_, Δρ_min_ (e Å^−3^)	0.40, −0.31

Computer programs: *APEX2* and *SAINT* (Bruker, 2014 ▸), *SHELXT* (Sheldrick, 2015*a* ▸), *SHELXL* (Sheldrick, 2015*b* ▸) and *OLEX2* (Dolomanov *et al.*, 2009 ▸).

## supplementary crystallographic information

### Crystal data

**Table:**

C_18_H_12_N_2_O_2_	*Z* = 4
*M**_r_* = 288.30	*F*(000) = 600
Triclinic, *P*1	*D*_x_ = 1.375 Mg m^−^^3^
*a* = 9.2660 (3) Å	Mo *K*α radiation, λ = 0.71073 Å
*b* = 12.9590 (4) Å	Cell parameters from 8081 reflections
*c* = 13.1010 (4) Å	θ = 2.6–25.7°
α = 96.2487 (15)°	µ = 0.09 mm^−^^1^
β = 109.1813 (15)°	*T* = 100 K
γ = 106.1061 (14)°	Block, white
*V* = 1392.39 (8) Å^3^	0.1 × 0.1 × 0.1 mm

### Data collection

**Table:**

Bruker APEXII CCD diffractometer	4470 reflections with *I* > 2σ(*I*)
φ and ω scans	*R*_int_ = 0.026
Absorption correction: multi-scan (SADABS; Bruker, 2014)	θ_max_ = 25.7°, θ_min_ = 1.7°
*T*_min_ = 0.628, *T*_max_ = 0.745	*h* = −11→11
17357 measured reflections	*k* = −15→15
5277 independent reflections	*l* = −15→15

### Refinement

**Table:**

Refinement on *F*^2^	Hydrogen site location: inferred from neighbouring sites
Least-squares matrix: full	H-atom parameters constrained
*R*[*F*^2^ > 2σ(*F*^2^)] = 0.041	*w* = 1/[σ^2^(*F*_o_^2^) + (0.0657*P*)^2^ + 0.2853*P*] where *P* = (*F*_o_^2^ + 2*F*_c_^2^)/3
*wR*(*F*^2^) = 0.115	(Δ/σ)_max_ = 0.001
*S* = 1.08	Δρ_max_ = 0.40 e Å^−^^3^
5277 reflections	Δρ_min_ = −0.31 e Å^−^^3^
398 parameters	Extinction correction: SHELXL, Fc^*^=kFc[1+0.001xFc^2^λ^3^/sin(2θ)]^-1/4^
0 restraints	Extinction coefficient: 0.026 (3)

### Special details

**Table:**

Geometry. All esds (except the esd in the dihedral angle between two l.s. planes) are estimated using the full covariance matrix. The cell esds are taken into account individually in the estimation of esds in distances, angles and torsion angles; correlations between esds in cell parameters are only used when they are defined by crystal symmetry. An approximate (isotropic) treatment of cell esds is used for estimating esds involving l.s. planes.

### Fractional atomic coordinates and isotropic or equivalent isotropic displacement parameters (Å^2^)

**Table:**

	*x*	*y*	*z*	*U*_iso_*/*U*_eq_	
O1	1.07827 (13)	0.32279 (9)	0.71767 (9)	0.0379 (3)	
O2	1.11420 (15)	0.18678 (11)	0.79164 (10)	0.0523 (3)	
N1	0.58804 (14)	−0.11853 (9)	0.53169 (10)	0.0267 (3)	
H1	0.5808	−0.1487	0.5876	0.032*	
N2	1.04678 (14)	0.22353 (11)	0.71390 (10)	0.0324 (3)	
C1	0.48711 (16)	−0.16207 (11)	0.42177 (12)	0.0258 (3)	
C2	0.35659 (17)	−0.26036 (11)	0.37438 (13)	0.0312 (3)	
H2	0.3235	−0.3083	0.4185	0.037*	
C3	0.27777 (17)	−0.28488 (12)	0.26075 (13)	0.0339 (3)	
H3	0.1894	−0.3517	0.2261	0.041*	
C4	0.32410 (17)	−0.21428 (12)	0.19502 (13)	0.0338 (3)	
H4	0.2663	−0.2334	0.1171	0.041*	
C5	0.45313 (17)	−0.11701 (12)	0.24236 (12)	0.0306 (3)	
H5	0.4844	−0.0691	0.1977	0.037*	
C6	0.53657 (16)	−0.09055 (11)	0.35684 (11)	0.0253 (3)	
C7	0.67397 (15)	0.00101 (11)	0.43280 (11)	0.0241 (3)	
C8	0.77372 (16)	0.09677 (11)	0.41936 (11)	0.0250 (3)	
H8	0.7534	0.1117	0.3475	0.030*	
C9	0.90234 (16)	0.17073 (11)	0.50954 (11)	0.0248 (3)	
C10	0.92260 (16)	0.14514 (11)	0.61431 (11)	0.0257 (3)	
C11	0.82673 (16)	0.05185 (11)	0.63224 (11)	0.0263 (3)	
H11	0.8457	0.0380	0.7044	0.032*	
C12	0.70141 (16)	−0.02055 (11)	0.53961 (11)	0.0240 (3)	
C13	1.01868 (16)	0.26510 (11)	0.49125 (11)	0.0255 (3)	
C14	0.96555 (18)	0.33925 (12)	0.43259 (12)	0.0314 (3)	
H14	0.8537	0.3314	0.4059	0.038*	
C15	1.0741 (2)	0.42439 (12)	0.41272 (14)	0.0377 (4)	
H15	1.0363	0.4747	0.3728	0.045*	
C16	1.2368 (2)	0.43656 (12)	0.45059 (14)	0.0389 (4)	
H16	1.3110	0.4949	0.4366	0.047*	
C17	1.29147 (18)	0.36317 (12)	0.50921 (13)	0.0355 (4)	
H17	1.4035	0.3716	0.5359	0.043*	
C18	1.18363 (17)	0.27788 (12)	0.52902 (12)	0.0297 (3)	
H18	1.2219	0.2275	0.5686	0.036*	
O3	0.39259 (13)	0.17339 (8)	0.23420 (9)	0.0372 (3)	
O4	0.54564 (13)	0.31480 (10)	0.36730 (9)	0.0424 (3)	
N3	0.55743 (14)	0.60781 (9)	0.13124 (10)	0.0289 (3)	
H3A	0.6607	0.6425	0.1717	0.035*	
N4	0.44465 (14)	0.27322 (10)	0.27258 (10)	0.0302 (3)	
C19	0.46298 (17)	0.64286 (11)	0.04645 (12)	0.0273 (3)	
C20	0.50540 (19)	0.73722 (11)	0.00593 (13)	0.0320 (3)	
H20	0.6134	0.7872	0.0348	0.038*	
C21	0.3850 (2)	0.75518 (12)	−0.07728 (13)	0.0345 (4)	
H21	0.4103	0.8199	−0.1050	0.041*	
C22	0.22615 (19)	0.68083 (12)	−0.12246 (13)	0.0345 (3)	
H22	0.1460	0.6956	−0.1802	0.041*	
C23	0.18495 (18)	0.58607 (12)	−0.08380 (12)	0.0307 (3)	
H23	0.0776	0.5351	−0.1153	0.037*	
C24	0.30359 (16)	0.56668 (11)	0.00213 (12)	0.0263 (3)	
C25	0.30496 (16)	0.48076 (11)	0.06358 (11)	0.0250 (3)	
C26	0.18845 (16)	0.38326 (11)	0.05811 (11)	0.0253 (3)	
H26	0.0807	0.3646	0.0062	0.030*	
C27	0.22709 (16)	0.31273 (11)	0.12733 (11)	0.0248 (3)	
C28	0.38882 (16)	0.34634 (11)	0.20367 (11)	0.0255 (3)	
C29	0.50785 (16)	0.44388 (11)	0.21468 (11)	0.0269 (3)	
H29	0.6141	0.4642	0.2692	0.032*	
C30	0.46465 (16)	0.51022 (11)	0.14253 (11)	0.0254 (3)	
C31	0.09599 (16)	0.21235 (11)	0.12135 (12)	0.0260 (3)	
C32	−0.01531 (18)	0.14815 (11)	0.01888 (13)	0.0328 (3)	
H32	−0.0024	0.1655	−0.0469	0.039*	
C33	−0.1454 (2)	0.05885 (12)	0.01173 (16)	0.0446 (4)	
H33	−0.2219	0.0162	−0.0587	0.054*	
C34	−0.1634 (2)	0.03214 (13)	0.10686 (18)	0.0467 (5)	
H34	−0.2524	−0.0288	0.1021	0.056*	
C35	−0.0517 (2)	0.09420 (13)	0.20895 (16)	0.0413 (4)	
H35	−0.0628	0.0746	0.2745	0.050*	
C36	0.07586 (18)	0.18444 (12)	0.21679 (13)	0.0319 (3)	
H36	0.1503	0.2278	0.2876	0.038*	

### Atomic displacement parameters (Å^2^)

**Table:**

	*U*^11^	*U*^22^	*U*^33^	*U*^12^	*U*^13^	*U*^23^
O1	0.0334 (6)	0.0313 (6)	0.0382 (6)	0.0010 (5)	0.0118 (5)	−0.0041 (5)
O2	0.0430 (7)	0.0552 (8)	0.0326 (6)	−0.0003 (6)	−0.0058 (5)	0.0137 (5)
N1	0.0263 (6)	0.0246 (6)	0.0297 (6)	0.0080 (5)	0.0110 (5)	0.0083 (5)
N2	0.0245 (6)	0.0380 (7)	0.0276 (7)	0.0035 (5)	0.0081 (5)	0.0029 (5)
C1	0.0225 (6)	0.0235 (7)	0.0321 (8)	0.0106 (5)	0.0095 (6)	0.0029 (5)
C2	0.0268 (7)	0.0229 (7)	0.0428 (9)	0.0080 (6)	0.0129 (7)	0.0047 (6)
C3	0.0251 (7)	0.0255 (7)	0.0429 (9)	0.0057 (6)	0.0084 (7)	−0.0036 (6)
C4	0.0282 (7)	0.0353 (8)	0.0307 (8)	0.0097 (6)	0.0067 (6)	−0.0046 (6)
C5	0.0287 (7)	0.0330 (8)	0.0290 (8)	0.0098 (6)	0.0113 (6)	0.0023 (6)
C6	0.0224 (7)	0.0242 (7)	0.0292 (7)	0.0090 (5)	0.0099 (6)	0.0021 (5)
C7	0.0214 (6)	0.0257 (7)	0.0259 (7)	0.0089 (5)	0.0097 (6)	0.0032 (5)
C8	0.0231 (7)	0.0276 (7)	0.0253 (7)	0.0088 (5)	0.0101 (6)	0.0058 (5)
C9	0.0214 (6)	0.0251 (7)	0.0288 (7)	0.0086 (5)	0.0102 (6)	0.0048 (5)
C10	0.0204 (6)	0.0280 (7)	0.0258 (7)	0.0071 (5)	0.0068 (6)	0.0022 (5)
C11	0.0254 (7)	0.0300 (7)	0.0245 (7)	0.0109 (6)	0.0091 (6)	0.0069 (6)
C12	0.0220 (6)	0.0229 (6)	0.0296 (7)	0.0096 (5)	0.0109 (6)	0.0058 (5)
C13	0.0244 (7)	0.0233 (7)	0.0266 (7)	0.0051 (5)	0.0104 (6)	0.0013 (5)
C14	0.0280 (7)	0.0295 (7)	0.0344 (8)	0.0087 (6)	0.0099 (6)	0.0057 (6)
C15	0.0440 (9)	0.0245 (7)	0.0432 (9)	0.0093 (7)	0.0159 (8)	0.0088 (6)
C16	0.0394 (9)	0.0221 (7)	0.0489 (10)	−0.0014 (6)	0.0201 (8)	0.0025 (6)
C17	0.0251 (7)	0.0303 (8)	0.0446 (9)	0.0017 (6)	0.0134 (7)	0.0005 (6)
C18	0.0264 (7)	0.0273 (7)	0.0337 (8)	0.0078 (6)	0.0111 (6)	0.0043 (6)
O3	0.0402 (6)	0.0291 (6)	0.0455 (7)	0.0135 (5)	0.0174 (5)	0.0120 (5)
O4	0.0354 (6)	0.0493 (7)	0.0313 (6)	0.0086 (5)	0.0023 (5)	0.0123 (5)
N3	0.0229 (6)	0.0228 (6)	0.0330 (7)	0.0011 (5)	0.0077 (5)	0.0008 (5)
N4	0.0258 (6)	0.0343 (7)	0.0316 (7)	0.0090 (5)	0.0122 (5)	0.0096 (5)
C19	0.0285 (7)	0.0231 (7)	0.0295 (7)	0.0068 (6)	0.0131 (6)	0.0003 (5)
C20	0.0357 (8)	0.0222 (7)	0.0390 (8)	0.0057 (6)	0.0196 (7)	0.0026 (6)
C21	0.0465 (9)	0.0258 (7)	0.0399 (9)	0.0143 (7)	0.0244 (7)	0.0099 (6)
C22	0.0418 (9)	0.0341 (8)	0.0337 (8)	0.0191 (7)	0.0159 (7)	0.0092 (6)
C23	0.0295 (7)	0.0288 (7)	0.0328 (8)	0.0098 (6)	0.0114 (6)	0.0039 (6)
C24	0.0270 (7)	0.0217 (7)	0.0300 (7)	0.0069 (5)	0.0129 (6)	0.0016 (5)
C25	0.0244 (7)	0.0229 (7)	0.0256 (7)	0.0069 (5)	0.0094 (6)	0.0004 (5)
C26	0.0211 (6)	0.0239 (7)	0.0264 (7)	0.0046 (5)	0.0072 (6)	0.0013 (5)
C27	0.0243 (7)	0.0233 (7)	0.0249 (7)	0.0056 (5)	0.0102 (6)	0.0006 (5)
C28	0.0261 (7)	0.0268 (7)	0.0243 (7)	0.0091 (6)	0.0104 (6)	0.0047 (5)
C29	0.0220 (7)	0.0278 (7)	0.0254 (7)	0.0057 (6)	0.0059 (6)	0.0001 (5)
C30	0.0231 (7)	0.0219 (6)	0.0279 (7)	0.0036 (5)	0.0104 (6)	−0.0004 (5)
C31	0.0237 (7)	0.0211 (6)	0.0346 (8)	0.0080 (5)	0.0126 (6)	0.0051 (5)
C32	0.0307 (8)	0.0245 (7)	0.0386 (8)	0.0079 (6)	0.0101 (7)	0.0019 (6)
C33	0.0321 (8)	0.0230 (8)	0.0640 (12)	0.0033 (6)	0.0085 (8)	−0.0033 (7)
C34	0.0356 (9)	0.0224 (8)	0.0860 (14)	0.0069 (7)	0.0295 (10)	0.0132 (8)
C35	0.0458 (9)	0.0322 (8)	0.0674 (12)	0.0200 (7)	0.0387 (9)	0.0226 (8)
C36	0.0335 (8)	0.0287 (7)	0.0408 (9)	0.0136 (6)	0.0199 (7)	0.0097 (6)

### Geometric parameters (Å, º)

**Table:**

O1—N2	1.2291 (17)	O3—N4	1.2296 (16)
O2—N2	1.2275 (17)	O4—N4	1.2319 (16)
N1—H1	0.8800	N3—H3A	0.8800
N1—C1	1.3829 (18)	N3—C19	1.3776 (19)
N1—C12	1.3734 (17)	N3—C30	1.3716 (17)
N2—C10	1.4610 (18)	N4—C28	1.4600 (18)
C1—C2	1.3966 (19)	C19—C20	1.394 (2)
C1—C6	1.409 (2)	C19—C24	1.4128 (19)
C2—H2	0.9500	C20—H20	0.9500
C2—C3	1.380 (2)	C20—C21	1.374 (2)
C3—H3	0.9500	C21—H21	0.9500
C3—C4	1.400 (2)	C21—C22	1.401 (2)
C4—H4	0.9500	C22—H22	0.9500
C4—C5	1.382 (2)	C22—C23	1.383 (2)
C5—H5	0.9500	C23—H23	0.9500
C5—C6	1.394 (2)	C23—C24	1.393 (2)
C6—C7	1.4462 (18)	C24—C25	1.4437 (19)
C7—C8	1.3940 (19)	C25—C26	1.3928 (18)
C7—C12	1.4113 (19)	C25—C30	1.4142 (19)
C8—H8	0.9500	C26—H26	0.9500
C8—C9	1.3890 (19)	C26—C27	1.3909 (19)
C9—C10	1.412 (2)	C27—C28	1.4120 (19)
C9—C13	1.4890 (19)	C27—C31	1.4875 (18)
C10—C11	1.3817 (19)	C28—C29	1.3846 (19)
C11—H11	0.9500	C29—H29	0.9500
C11—C12	1.3867 (19)	C29—C30	1.383 (2)
C13—C14	1.390 (2)	C31—C32	1.389 (2)
C13—C18	1.3975 (19)	C31—C36	1.394 (2)
C14—H14	0.9500	C32—H32	0.9500
C14—C15	1.384 (2)	C32—C33	1.389 (2)
C15—H15	0.9500	C33—H33	0.9500
C15—C16	1.380 (2)	C33—C34	1.378 (3)
C16—H16	0.9500	C34—H34	0.9500
C16—C17	1.386 (2)	C34—C35	1.379 (3)
C17—H17	0.9500	C35—H35	0.9500
C17—C18	1.381 (2)	C35—C36	1.379 (2)
C18—H18	0.9500	C36—H36	0.9500
			
C1—N1—H1	125.5	C19—N3—H3A	125.5
C12—N1—H1	125.5	C30—N3—H3A	125.5
C12—N1—C1	109.05 (11)	C30—N3—C19	109.05 (11)
O1—N2—C10	119.42 (12)	O3—N4—O4	122.28 (12)
O2—N2—O1	122.57 (13)	O3—N4—C28	119.53 (12)
O2—N2—C10	118.00 (13)	O4—N4—C28	118.17 (12)
N1—C1—C2	129.30 (13)	N3—C19—C20	129.19 (13)
N1—C1—C6	109.00 (12)	N3—C19—C24	109.16 (12)
C2—C1—C6	121.70 (13)	C20—C19—C24	121.64 (14)
C1—C2—H2	121.4	C19—C20—H20	121.3
C3—C2—C1	117.14 (14)	C21—C20—C19	117.45 (14)
C3—C2—H2	121.4	C21—C20—H20	121.3
C2—C3—H3	119.0	C20—C21—H21	119.1
C2—C3—C4	121.97 (13)	C20—C21—C22	121.89 (14)
C4—C3—H3	119.0	C22—C21—H21	119.1
C3—C4—H4	119.7	C21—C22—H22	119.7
C5—C4—C3	120.65 (14)	C23—C22—C21	120.61 (15)
C5—C4—H4	119.7	C23—C22—H22	119.7
C4—C5—H5	120.6	C22—C23—H23	120.6
C4—C5—C6	118.76 (14)	C22—C23—C24	118.85 (14)
C6—C5—H5	120.6	C24—C23—H23	120.6
C1—C6—C7	106.36 (12)	C19—C24—C25	106.21 (12)
C5—C6—C1	119.77 (13)	C23—C24—C19	119.53 (13)
C5—C6—C7	133.86 (14)	C23—C24—C25	134.26 (13)
C8—C7—C6	133.64 (13)	C26—C25—C24	133.90 (13)
C8—C7—C12	119.83 (12)	C26—C25—C30	119.64 (13)
C12—C7—C6	106.53 (12)	C30—C25—C24	106.46 (12)
C7—C8—H8	119.5	C25—C26—H26	119.4
C9—C8—C7	120.95 (13)	C27—C26—C25	121.17 (12)
C9—C8—H8	119.5	C27—C26—H26	119.4
C8—C9—C10	116.54 (12)	C26—C27—C28	116.44 (12)
C8—C9—C13	119.74 (12)	C26—C27—C31	118.77 (12)
C10—C9—C13	123.46 (12)	C28—C27—C31	124.67 (12)
C9—C10—N2	119.60 (12)	C27—C28—N4	120.38 (12)
C11—C10—N2	115.52 (12)	C29—C28—N4	114.84 (12)
C11—C10—C9	124.79 (12)	C29—C28—C27	124.63 (13)
C10—C11—H11	121.7	C28—C29—H29	121.6
C10—C11—C12	116.66 (13)	C30—C29—C28	116.83 (12)
C12—C11—H11	121.7	C30—C29—H29	121.6
N1—C12—C7	109.05 (12)	N3—C30—C25	109.12 (12)
N1—C12—C11	129.75 (13)	N3—C30—C29	129.64 (12)
C11—C12—C7	121.20 (12)	C29—C30—C25	121.24 (12)
C14—C13—C9	121.11 (12)	C32—C31—C27	119.84 (13)
C14—C13—C18	118.63 (13)	C32—C31—C36	118.71 (13)
C18—C13—C9	120.21 (12)	C36—C31—C27	121.33 (13)
C13—C14—H14	119.7	C31—C32—H32	119.7
C15—C14—C13	120.60 (14)	C33—C32—C31	120.58 (15)
C15—C14—H14	119.7	C33—C32—H32	119.7
C14—C15—H15	119.8	C32—C33—H33	120.0
C16—C15—C14	120.38 (15)	C34—C33—C32	120.01 (16)
C16—C15—H15	119.8	C34—C33—H33	120.0
C15—C16—H16	120.2	C33—C34—H34	120.1
C15—C16—C17	119.61 (14)	C33—C34—C35	119.77 (15)
C17—C16—H16	120.2	C35—C34—H34	120.1
C16—C17—H17	119.9	C34—C35—H35	119.7
C18—C17—C16	120.26 (14)	C34—C35—C36	120.60 (16)
C18—C17—H17	119.9	C36—C35—H35	119.7
C13—C18—H18	119.7	C31—C36—H36	119.8
C17—C18—C13	120.52 (14)	C35—C36—C31	120.31 (15)
C17—C18—H18	119.7	C35—C36—H36	119.8
			
O1—N2—C10—C9	33.96 (18)	O3—N4—C28—C27	35.93 (18)
O1—N2—C10—C11	−142.78 (13)	O3—N4—C28—C29	−139.86 (13)
O2—N2—C10—C9	−147.07 (14)	O4—N4—C28—C27	−145.47 (13)
O2—N2—C10—C11	36.19 (18)	O4—N4—C28—C29	38.74 (17)
N1—C1—C2—C3	−178.83 (13)	N3—C19—C20—C21	−177.36 (13)
N1—C1—C6—C5	179.82 (11)	N3—C19—C24—C23	178.82 (12)
N1—C1—C6—C7	−0.43 (14)	N3—C19—C24—C25	−0.84 (15)
N2—C10—C11—C12	176.07 (11)	N4—C28—C29—C30	173.21 (11)
C1—N1—C12—C7	0.29 (14)	C19—N3—C30—C25	−0.34 (15)
C1—N1—C12—C11	−179.86 (13)	C19—N3—C30—C29	178.60 (13)
C1—C2—C3—C4	−0.8 (2)	C19—C20—C21—C22	−1.6 (2)
C1—C6—C7—C8	−179.68 (13)	C19—C24—C25—C26	−178.57 (14)
C1—C6—C7—C12	0.60 (14)	C19—C24—C25—C30	0.62 (14)
C2—C1—C6—C5	0.67 (19)	C20—C19—C24—C23	−0.4 (2)
C2—C1—C6—C7	−179.58 (12)	C20—C19—C24—C25	179.97 (12)
C2—C3—C4—C5	0.7 (2)	C20—C21—C22—C23	0.3 (2)
C3—C4—C5—C6	0.1 (2)	C21—C22—C23—C24	1.0 (2)
C4—C5—C6—C1	−0.78 (19)	C22—C23—C24—C19	−1.0 (2)
C4—C5—C6—C7	179.56 (14)	C22—C23—C24—C25	178.56 (14)
C5—C6—C7—C8	0.0 (3)	C23—C24—C25—C26	1.8 (3)
C5—C6—C7—C12	−179.70 (14)	C23—C24—C25—C30	−178.97 (15)
C6—C1—C2—C3	0.13 (19)	C24—C19—C20—C21	1.7 (2)
C6—C7—C8—C9	−178.43 (13)	C24—C25—C26—C27	177.60 (13)
C6—C7—C12—N1	−0.56 (14)	C24—C25—C30—N3	−0.18 (14)
C6—C7—C12—C11	179.58 (11)	C24—C25—C30—C29	−179.23 (12)
C7—C8—C9—C10	−1.82 (18)	C25—C26—C27—C28	0.96 (19)
C7—C8—C9—C13	172.60 (12)	C25—C26—C27—C31	177.21 (12)
C8—C7—C12—N1	179.68 (11)	C26—C25—C30—N3	179.14 (11)
C8—C7—C12—C11	−0.18 (19)	C26—C25—C30—C29	0.09 (19)
C8—C9—C10—N2	−174.94 (11)	C26—C27—C28—N4	−174.31 (12)
C8—C9—C10—C11	1.5 (2)	C26—C27—C28—C29	1.0 (2)
C8—C9—C13—C14	55.80 (18)	C26—C27—C31—C32	44.14 (18)
C8—C9—C13—C18	−121.42 (14)	C26—C27—C31—C36	−131.90 (14)
C9—C10—C11—C12	−0.5 (2)	C27—C28—C29—C30	−2.4 (2)
C9—C13—C14—C15	−177.70 (13)	C27—C31—C32—C33	−175.23 (13)
C9—C13—C18—C17	177.90 (13)	C27—C31—C36—C35	176.57 (13)
C10—C9—C13—C14	−130.18 (14)	C28—C27—C31—C32	−139.94 (14)
C10—C9—C13—C18	52.60 (18)	C28—C27—C31—C36	44.02 (19)
C10—C11—C12—N1	179.96 (12)	C28—C29—C30—N3	−177.09 (13)
C10—C11—C12—C7	−0.20 (18)	C28—C29—C30—C25	1.75 (19)
C12—N1—C1—C2	179.16 (13)	C30—N3—C19—C20	179.86 (13)
C12—N1—C1—C6	0.10 (14)	C30—N3—C19—C24	0.75 (15)
C12—C7—C8—C9	1.25 (19)	C30—C25—C26—C27	−1.50 (19)
C13—C9—C10—N2	10.87 (19)	C31—C27—C28—N4	9.68 (19)
C13—C9—C10—C11	−172.72 (12)	C31—C27—C28—C29	−174.96 (12)
C13—C14—C15—C16	0.3 (2)	C31—C32—C33—C34	−1.1 (2)
C14—C13—C18—C17	0.6 (2)	C32—C31—C36—C35	0.5 (2)
C14—C15—C16—C17	−0.3 (2)	C32—C33—C34—C35	−0.1 (2)
C15—C16—C17—C18	0.4 (2)	C33—C34—C35—C36	1.5 (2)
C16—C17—C18—C13	−0.6 (2)	C34—C35—C36—C31	−1.7 (2)
C18—C13—C14—C15	−0.4 (2)	C36—C31—C32—C33	0.9 (2)

### Hydrogen-bond geometry (Å, º)

**Table:**

*D*—H···*A*	*D*—H	H···*A*	*D*···*A*	*D*—H···*A*
N1—H1···O3^i^	0.88 (1)	2.33 (1)	3.1825 (16)	162 (1)
N1—H1···O4^i^	0.88 (1)	2.38 (1)	3.1331 (17)	143 (1)
N1—H1···N4^i^	0.88 (1)	2.59 (1)	3.4610 (17)	168 (1)
N3—H3*A*···O1^ii^	0.88 (1)	2.26 (1)	3.1079 (18)	162 (1)
N3—H3*A*···O2^ii^	0.88 (1)	2.45 (1)	3.2039 (19)	143 (1)
N3—H3*A*···N2^ii^	0.88 (1)	2.60 (1)	3.4700 (19)	170 (1)

Symmetry codes: (i) −*x*+1, −*y*, −*z*+1; (ii) −*x*+2, −*y*+1, −*z*+1.
